# Effect of arch wires and brackets in orthodontics for releasing nickel ions

**DOI:** 10.6026/973206300210035

**Published:** 2025-01-31

**Authors:** Osric D Costa, Sunaina D., Praveen Kumar Gonuguntla Kamma, Abhilasha Mishra, Anurag Sahu, Ruchi Patel

**Affiliations:** 1 Department of Orthodontics and Dentofacial Orthopedics, Braces Plus Complete Dental Care, Bandra East, Mumbai, Maharashtra, India; 2 Department of Dentistry, Datia district Hospital, Madhya Pradesh - 475661, India; 3 Dentist, Texas State Dental Association, US; 4 Department of Orthodontics and Dentofacial Orthopaedics, Rishiraj College of Dental Science and Research Center, Bhopal, Madhya Pradesh, India; 5 Department of Orthodontics and Dentofacial Orthopaedics Hitkarini Dental College & Hospital, Jabalpur, Madhya Pradesh, India; 6 Department of Prosthodontics, Karnavati School of Dentistry, Karnavati University, Gandhinagar, Gujarat, India

**Keywords:** Ni ion, release, orthodontic archwire, orthodontic brackets

## Abstract

The amount of nickel ions released from orthodontic wires and brackets is of interest to dentists. The amount of nickel ions emitted from a combination of one of the five arch wires (NiTi, SS, Cu NiTi, Co-Cr-Ni alloys and ion implanted NiTi), five orthodontic brackets and one band was investigated. The wire was 0.016 inches in length. Data shows the release of Ni ion from assembly of orthodontic archwire, orthodontic brackets and orthodontic band in all categories at all-time checkpoints with maximum increase in release of Ni from baselines to 7^th^ day. Thereafter, release of Ni decreased as the time duration increased in all categories. Hence, there is significant release of Ni ion from orthodontic archwire and orthodontic brackets. Thus, orthodontic therapy for patients who are Ni sensitive may be difficult.

## Background:

Nowadays, a key prerequisite for effective clinical conduct in the oral cavity is the biocompatibility of dental materials. It incorporates information from engineering, clinical experience, biology and patient risk factors [1, 2-3]. When assessing the biological compatibility of any kind of dental material, two primary variables seem to be crucial: surface features and different forms of corrosion or breakdown of the material [2, 3-4]. Physiological fluids and dental materials in the mouth are constantly interacting [4, 5-6]. Saliva acts as a hypotonic fluid that contains proteins, nitrogenous substances, potassium, chloride and sodium and bio actonate. When orthodontic fixtures are exposed to the harsh electrolytic environment of the human mouth, corrosion-the progressive deterioration of materials by electrolysis attack-becomes a significant concern [5, 6-7]. Stainless steel (SS), nickel-titanium alloys (NiTi), cobalt-chromium-nickel alloys (CoCrNi), β-titanium alloys and other wire and bracket types are used to correct malocclusion [8, 9- 10]. In orthodontic appliances, alloys like nickel-titanium and stainless steel are frequently utilized. Ion release is facilitated by the corrosion that occurs when metals are subjected to heat and chemical transformations in the mouth cavity [11, 12-13]. Ions like nickel are released during this process and when they surpass a certain threshold, they can have toxic and physiologic effects like soft tissue colouring and allergic reactions [14, 15- 16]. One of the things that causes corrosion in the mouth cavity is food and beverages. The consumption of caffeinated and acidic beverages, including tea, coffee and soft drinks, is increasing at the moment [12, 13, 14- 15]. Phosphoric acid, methyl-xanthine, polyphenol, maleic acid, lactic acid, tartaric acid and citric acid are all present in these beverages. Acidic diets and beverages impair the lifespan of dental restorations in addition to damaging enamel [11, 12-13]. These beverages cause metal corrosion and erode the substances' solubility, surface integrity and hardness [14, 15-16]. A thorough analysis of common orthodontic alloys under various environmental circumstances is crucial given the serious ramifications of ion emission and its possible effects on biological structures [9, 10, 11- 12]. Both corrosion behaviors including the measurement of released ions should be included in this assessment [13, 14-15]. Prior research has evaluated the ion discharge from alloys in a variety of water-based settings, which includes saliva and mouthwash. Nevertheless, little is known about the acidic environment that soft drinks create [16, 17-18]. Therefore, it is crucial to evaluate the potential impact of soft drinks on the orthodontic therapeutic appliances given the rise in the intake of soft drinks and the propensity for orthodontic treatment [18, 19-20]. The potential risk of arch wire corrosion while employing nickel titanium (NiTi) arch wire for orthodontic dental treatment stems from the detrimental biological consequences of the liberated Ni ion [21, 22, 23-24]. Thus, a high level of corrosion resistance in NiTi arch wire is essential to its biocompatibility. However, NiTi arch wires' surface corrosion may make the friction at the bracket-arch wire contact higher, which would decrease the free sliding movement during orthodontic therapy [25, 26-27]. Therefore, it is of interest to evaluate the amount of nickel ions released from orthodontic wires and brackets.

## Methods and Materials:

It was an *in vitro* study in which, the amount of nickel ions emitted from a combination of five different types of arch wires (NiTi, SS, Cu NiTi, Co-Cr-Ni alloys and ion implanted NiTi), five orthodontic brackets and one band (American Orthodontics, Sheboygan, USA) was investigated. The wire was 0.016 inches in length (Table 1). Each sample was placed in a different polyethylene screw-top bottle with 100 millilitres of artificial saliva in order to measure the Ni release. 0.4 G of sodium chloride, 1.21 G of potassium chloride, 0.78 G of sodium hypophosphate, 0.005 G of sodium sulphide, 1 G of urea and 1000 ml of distilled and deionized water made up the stimulated saliva medium. The pH of artificial saliva was tested using E. Merch (D-6100 Darmstadt F.R. Germany) pH indicator papers with a high degree of sensitivity (0.2 units sensitivity) after 50 ml increments of 10N sodium hydroxide were added to get the pH down to 6.75 ± 0.15. An atomic absorption spectrophotometer model, which is based on the distinct spectrum of each element, was used to conduct the analysis. By measuring the absorbed radiation, an atomic absorption spectrometer determines the amounts of chemical components found in environmental samples. Reading the spectra generated when the sample is excited by radiation is how this is accomplished. The quantity of energy in the form of photons of light that are absorbed by the sample is measured using atomic absorption techniques. A discharge lamp (hollow cathode lamp) produces distinctive wavelengths for each element under analysis, which are then absorbed by the element's cloud or vapor. Since the equipment's sensitivity was limited to 1 ppm", a "standard addition method" was employed. The produced samples were evaluated on the seventh, fourteenth and twenty-first days after being submerged in artificial saliva at 36.5° and pH 5.6-7. An atomic adsorption spectrophotometer was used to measure the amount of Ni and Ti ions emitted from NiTi wires in a 20 ml volume of known concentration. They plot a conventional graph. 10 ml of a saliva test sample is mixed with 10 ml of a known concentration of Ni. When the values for the released metal dropped, it was evident that the standard solution was diluted due to the deionized water displaying a lower ppm level. In order to get correct results, each sample was evaluated three times and the average was calculated. The data were then statistically examined".

## Results:

## Statistical analysis:

One-way analysis of variance (ANOVA) was used to compare the findings of the solution's absorption analysis. Table 2 displays the findings of a one-way ANOVA used to compare the average daily Ni release across the various groups. Turkey's honestly significant difference (HSD) was used to examine the mean difference using the multiple comparison test and a difference was deemed significant at P>0.05. version 16.0 of the statistical software SPSS for Windows. For statistical analysis and data processing, SPSS Inc. of the United States of America, Chicago, was utilized. In our study there was release of Ni ion from assembly of orthodontic archwire, orthodontic brackets and orthodontic band in all categories at all-time checkpoints with maximum increase in release of Ni between baselines to 7^th^ day. Thereafter, release of Ni decreased as the time duration increased in all categories. As far as comparison between different categories are concerned regarding release of Ni release then there was minimum release of Ni from assembly consisting SS wires followed by assembly consisting of Ion implanted NiTi wire. The Ni release was maximum in assembly consisting Co-Cr-Ni wire followed by assembly consisting of NiTi wire and CuNiTi wires. The findings were significant statistically (Table 2, Table 3).

It was observed that mean increase in Ni release in category 1 specimens (one SS archwire + 5 orthodontic brackets + 1 orthodontic band) was 6.64 ± 0.52 µg, 0.68±0.21 µg, 2.01±0.34 µg and 1.53±0.55 µg from baseline till 7th day follow up, 7^th^ day to 14^th^ day, 7^th^ day to 21^st^ day and 14^th^ day to 21^st^ day. The findings were significant statistically with increase in Ni release at different time intervals. Similarly, there was increase in Ni release at different time intervals in group 2 specimens comprising one NiTi wire + 5 orthodontic brackets + one orthodontic band. Mean increase in Ni release from baseline till 7^th^ day follow up, 7^th^ day to 14^th^ day, 7^th^ day to 21^st^ day and 14^th^ day to 21^st^ day was 7.45 ± 0.49 µg, 1.46±0.71 µg, 4.51±0.57 µg, 3.25±0.16 µg. Mean increase in Ni release in category 3 specimens comprising of 1 Cu NiTi wire + 5 orthodontic brackets + One orthodontic band was 6.23 ± 0.52 µg, 1.35±0.59 µg, 3.06±0.66 µg and 1.82±0.31 µg from baseline till 7^th^ day follow up, 7^th^ day to 14^th^ day, 7^th^ day to 21^st^ day and 14^th^ day to 21^st^ day. The findings were significant statistically with increase in Ni release at different time intervals. There was increase in Ni release at different time intervals in group 4 specimens comprising one Co-Cr-Ni wire + 5 orthodontic brackets + one orthodontic band. Mean increase in Ni release from baseline till 7^th^ day follow up, 7^th^ day to 14^th^ day, 7^th^ day to 21^st^ day and 14^th^ day to 21^st^ day was 7.14 ± 0.59 µg, 1.97±0.49 µg, 3.01±0.47 µg, 1.44±0.35 µg. The mean increase in Ni release in category 5 specimens (one archwire + 5 orthodontic brackets + 1 orthodontic band) was 6.64 ± 0.41 µg, 0.98±0.37µg, 2.34±0.34 µg and 1.73±0.42 µg from baseline till 7^th^ day follow up, 7^th^ day to 14^th^ day, 7^th^ day to 21^st^ day and 14^th^ day to 21^st^ day. The findings were significant statistically with increase in Ni release at different time intervals (Table 2, Table 3).

## Discussion:

Alloys containing nickel (Ni) are now a necessary component of practically all common orthodontic procedures. In order to produce tooth movement, modern orthodontics uses a variety of bonded attachments, arch wires and other tools [10, 11-12]. They are subjected to a variety of stresses in oral surroundings, including as masticatory force, temperature changes, immersion in saliva and ingested fluids, which results in complex demands [11, 12-13]. The 1930s saw the introduction of orthodontic appliances, such as arch wires, brackets and bands [14, 15-16]. Since then, the alloys-which are composed of stainless steel with 8-12% Ni, 17-22% chromium and different amounts of manganese, copper, titanium and iron-have grown to be an indispensable component in orthodontics [11, 12- 13]. These are reasonably priced and incredibly durable. The close closeness and unfavourable circumstances of the alloy materials cause corrosion and unfavourable biological reactions, as well as increased mechanical friction [12, 13, 14- 15]. The potential risk of arch wire corrosion while employing nickel titanium (NiTi) arch wire for orthodontic dental treatment stems from the detrimental biological consequences of the liberated Ni ion [15, 16-17]. Thus, a high level of corrosion resistance in NiTi arch wire is essential to its biocompatibility. However, NiTi arch wires' surface corrosion may make the friction at the bracket-arch wire contact higher, which would decrease the free sliding movement during orthodontic therapy [18, 19, 20-21]. The aim of this study was to evaluate the amount of nickel ions released from orthodontic wires and brackets. The findings of present study are having similarity with findings of other research which also found Ni ion release from the orthodontic archwires and orthodontic brackets [25, 26-27]. Some studies also found that Ni release was greater in initial days of experiment as compared to the later phases of experiment [24, 25, 26, 27-28]. These findings are similar to the findings of present study. Surface characteristics and various types of corrosion or material breakdown appear to be the two main factors that are most important when evaluating the biological compatibility of any type of dental material [18, 19, 20-21]. In the mouth, dental materials and physiological fluids interact all the time. With its proteins, nitrogenous materials, potassium, chloride, sodium and bioactonate, saliva functions as a hypotonic fluid [22, 23- 24].

Corrosion or the progressive degradation of materials by electrolysis assault becomes a serious issue when orthodontic fixtures are exposed to the hostile electrolytic environment of the human mouth [11, 12- 13]. The findings of present study are similar to the findings of some other research that also found that minimum Ni release is observed in SS orthodontic archwire [23, 24, 25-26]. Some studies also found that ion implanted NiTi wire had Ni release lesser than other Ni orthodontic archwires [27- 28]. To rectify malocclusion, many wire and bracket types are utilized, including stainless steel, nickel-titanium alloys, cobalt-chromium-nickel alloys and β-titanium alloys [13, 14-15]. Alloys like as stainless steel and nickel-titanium are commonly used in orthodontic appliances. When metals undergo thermal and chemical changes in the oral cavity, corrosion takes place, which facilitates ion release [16, 17, 18- 19]. This process releases ions like nickel, which can have physiologic and toxic effects such soft tissue coloration and allergic reactions when they reach a particular threshold [20, 21, [22-23]. Foods and drinks are among the items that corrode the oral cavity. Currently, there is a rise in the intake of acidic and caffeinated liquids, such as tea, coffee and soft drinks [14, 15- 16]. Numerous publications have documented *in vitro* corrosion of orthodontic appliances; however, comparisons between the research are not consistent because to differences in study designs and other electrochemical variables [12, 13, 14, 15-16]. Technique-sensitive preparation and analytical methods can also lead to variation; some corrosion products may stick to the metal surface and not be accessible for the solutes' experimental analysis, going unnoticed. According to several studies, simulated whole mouth orthodontic equipment released 20 µg of Ni daily [15, 16, 17- 18]. The overall Ni release in this study was significantly less than the typical daily consumption of 200-300 µg of Ni. However, because the quantity of Ni needed to produce contact hypersensitivity reactions varies from person to person, the levels cannot be directly compared [16, 17- 18-19]. According to a study, a Ni-sensitive patient experienced significant allergic reactions following the insertion of a NiTi arch wire [12, 13-14]. The detrimental consequences of discharged Ni *in vitro* cell cultures and the extent to which patients actually absorb the corrosive compounds need to be investigated further [14, 15, 16- 17]. Ni-sensitive patients can now benefit from the use of Ni-free brackets, such as ceramic and titanium brackets. Untreated NiTi wires can be substituted with implanted NiTi among the arch wires [21, 22, 23-24].

## Conclusion:

Data shows that there is a significant release of Ni ion from orthodontic archwires and orthodontic brackets. Hence, orthodontic therapy for patients who are Ni sensitive may be difficult.

## Figures and Tables

**Table 1:** Details about the study specimens

**Category**	**Description of specimen**	**Number**
1	SS wire + 5 orthodontic brackets + One orthodontic band	24
2	NiTi wire + 5 orthodontic brackets + One orthodontic band	24
3	Cu NiTi wire + 5 orthodontic brackets + One orthodontic band	24
4	Co-Cr-Ni wire + 5 orthodontic brackets + One orthodontic band	24
5	Ion implanted NiTi wire + 5 orthodontic brackets + One orthodontic band	24

**Table 2:** Mean change at different time intervals in different categories

	**Baseline to 7^th^ day**	**7^th^ day to 14^th^ day**	**7^th^ day to 21^st^ day**	**14^th^ day to 21^st^ day**
**Category 1**				
Change mean ±SD (µg)	6.64 ± 0.52	0.68±0.21	2.01±0.34	1.53±0.55
P value (significant)	<0.001			
Category 2				
Change mean ± SD (µg)	7.45 ± 0.49	1.46±0.71	4.51±0.57	3.25±0.16
Category 3				
Change mean ± SD (µg)	6.23 ± 0.52	1.35±0.59	3.06±0.66	1.82±0.31
P value (significant)	<0.001			
Category 4				
Change mean ± SD (µg)	7.14 ± 0.59	1.97±0.49	3.01±0.47	1.44±0.35
P value (significant)	<0.001			
Category 5				
Change mean ± SD (µg)	6.64 ± 0.41	0.98±0.37	2.34±0.34	1.73±0.42
P value (significant)	<0.001			

**Table 3:** Compare the average per day nickel release between different groups

	**Variations between different categories**	**Variations within categories**
df	3	4.5
Sum of squares	0.564	0.053
Mean squares	0.188	0.001
F ratio	157.88	
P value	< 0.0001 (significant)	
